# Application of bronchoscopy in the diagnosis and treatment of peripheral pulmonary lesions in China: a national cross-sectional study

**DOI:** 10.7150/jca.84220

**Published:** 2023-05-15

**Authors:** Haidong Huang, Ning Wu, Sen Tian, Dongchen Shi, Changhui Wang, Guangfa Wang, Faguang Jin, Shiyue Li, Yuchao Dong, Michael J Simoff, Qiang Li, Chong Bai

**Affiliations:** 1 Department of Respiratory and Critical Care Medicine, The First Affiliated Hospital of Naval Medical University, Shanghai, China.; 2 Department of Respiratory Medicine, Shanghai Tenth People's Hospital, Tongji University School of Medicine, Shanghai, China.; 3 Department of Respiratory and Critical Care Medicine, Peking University First Hospital, Beijing, China.; 4 Department of Respiratory and Critical Care Medicine, Tangdu Hospital, Air Force Military Medical University, Xian, China.; 5 Guangzhou Institute of Respiratory Health, State Key Laboratory of Respiratory Disease, the First Affiliated Hospital of Guangzhou Medical University, Guangzhou, China.; 6 Department of Pulmonary and Critical Care Medicine, Henry Ford Hospital, Detroit, MI, USA.; 7 Department of Pulmonary and Critical Care Medicine, Shanghai East Hospital, Tongji University School of Medicine, Shanghai, China.

**Keywords:** bronchoscopy, peripheral pulmonary lesions, endobronchial ultrasound, navigational bronchoscopy, diagnosis, treatment

## Abstract

**Background:** Bronchoscopy has gradually become valuable armamentarium in evaluating and applying endoscopic therapy to peripheral pulmonary lesions (PPLs) around the world. We aimed to make a comprehensive understanding of the application of bronchoscopy in the diagnosis and treatment of PPLs in China.

**Methods:** A cross-sectional survey was carried out in China between January 2022 and March 2022. The survey was in the form of an online questionnaire which was filled in with real-time data by the respondents.

**Results:** A total of 347 doctors from 284 tertiary hospitals (81.8%) and 63 secondary general hospitals (18.2%) were included in the data analysis. More than half of the surveyed doctors (55.0%) had independently performed respiratory endoscopy for 5-15 years. Higher proportions of hospitals with a fixed nursing team, anesthesiologists and rapid on-site evaluation (ROSE) during bronchoscopic procedures were performed in tertiary hospitals than those in secondary general hospitals (P<0.001 each). There were 316 hospitals (91.7%) eligible for performing biopsies of PPLs less than 30mm, while more than 300 PPLs biopsies were performed in only 78 hospitals (24.7%) per year. Radial probe endobronchial ultrasound (r-EBUS) (50.3%) was the commonest type of technique used in the guidance of a bronchoscope to PPLs, followed by navigational bronchoscopy (30.3%) and cone beam CT (CBCT) (17.0%). Although two thirds of the surveyed hospitals had at least one bronchoscopic guidance devices, the actual utilization of these devices was not high due to high capital costs and absence of training. To note, more diagnostic procedures and allocated devices were concentrated in the southeast region and coastal cities. Furthermore, therapeutic bronchoscopic interventions for peripheral lung cancer and/or high-risk PPLs could be performed in 124 (35.7%) of the 347 involved hospitals.

**Conclusions:** Bronchoscopy for the diagnosis of PPLs has been carried out in most hospitals in China and yields in different hospitals and regions varied greatly. To date, only a few hospitals in China can develop therapeutic bronchoscopy for PPLs.

## Introduction

There has been an increasing number of solitary pulmonary nodules (SPNs) that remain to be evaluated due to the rise in the clinical use of chest computed tomography (CT) screening, especially low-dose CT 1. According to the randomized NELSON trial 2, over 70% of SPNs develop in the periphery of the lungs. The likelihood of malignancy for peripheral pulmonary lesions (PPLs) between 8 and 20 mm was reported to be about 18%, and for lesions between 20 and 30 mm, about 50% 3. Given the possible presence of lung cancer, the detection of PPLs frequently requires tissue diagnosis for further patient management 4. Whilst CT-guided transthoracic biopsies have been documented to be a well-established and highly accurate interventional diagnostic procedure 5, there has been concern regarding the exposure to radiation and a considerable complication rate (e.g., bleeding and pneumothorax) 6, 7, which has hampered further development of this technique. By comparison, multiple advanced bronchoscopic navigational techniques such as radial probe endobronchial ultrasound (r-EBUS), virtual bronchoscopic navigation (VBN) and electromagnetic navigation bronchoscopy (ENB), and bronchoscopic imaging techniques including confocal laser endomicroscopy and Raman spectroscopy serve as minimally invasive procedures for the diagnosis of PPLs 8-10. Furthermore, endoscopic technology evolved in the past decades and bronchoscopy also has potential to be an alternative to open surgical treatments of PPLs, particularly for patients with significant medical comorbidities or poor performance status 4, 11, 12. There already have been international reports on the diagnosis and treatment of respiratory endoscopy in many countries 13-20; however, rare clinical studies concerning the application of bronchoscopy in the management of PPLs have been involved. Therefore, this study was performed with network questionnaires nationwide in 2022 in order to make a comprehensive understanding of clinical practice of bronchoscopy in the diagnosis and treatment of PPLs in China. In this way, we can better promote the widespread adoption of bronchoscopy in the management of PPLs.

## Methods

### Trial design and oversight

This cross-sectional nationwide survey was conducted from January 2022 to March 2022 by both Respiratory endoscopy branch of Chinese Endoscopist Association, Chinese Medical Doctor Association (CMDA) and Interventional Pulmonology branch of Chinese Association of Chest Physicians, CMDA. The survey form was structured and consisted of 25 questions related to bronchoscopic practices divided into four sections: 1) General information; 2) Personnel of respiratory endoscopy team, such as nursing, anesthesia and pathology personnel; 3) Application of diagnostic bronchoscopy procedures in 2021 and 4) Therapeutic bronchoscopic interventions. The questionnaire was anonymous and no reminders were sent. The questions were of either a descriptive response type or single/multiple option type. The option type questions either had a “Yes/No” response or option for multiple responses, such as “always, most of the times, sometimes, and never” wherever considered appropriate. Details of questionnaire are listed in Table ​1.

The survey was in the form of an online questionnaire which was filled in with real-time data by the respondents. Questionnaires that didn't conform to the standards or failed to fill in data were regarded as invalid. The survey was distributed across 29 provinces and 137 cities and all classes of hospitals were involved. If more than one doctor responded in a hospital, we only kept the answer from the doctor who had been independently engaged in respiratory interventions the longest for final data analysis because they had a better understand about bronchoscopic practices of their hospitals. No financial incentive was offered to the participants for responding in this survey.

### Statistical analysis

Statistical analysis of the data was performed using the software package IBM SPSS Statistics 26.0 (Statistical Package for the Social Sciences). Continuous parameters are presented by means when normally distributed or medians and standard deviations or range. Categorical variables are reported as frequencies and percentages. Univariate analysis involved the use of analysis of variance (ANOVA) for multiple continuous variables. For the analysis of correlation, the chi-square test was used for categorical data. Values were considered statistically significant for a level of P < 0.05.

## Results

### General information

Over 700 doctors from 347 hospitals in 137 cities in 29 provinces across the country completed the questionnaire. Finally, 347 valid responses from different hospitals were screened for data analysis. Among the 347 hospitals, there were 284 tertiary hospitals (81.8%), of which 276 (79.5%) were tertiary general hospitals and 8 (2.3%) were chest or pulmonary specialist hospitals. The other 63 hospitals (18.2%) were secondary general hospitals.

### Personnel of respiratory endoscopy team

#### Endoscopists

Among the 347 doctors included in the data analysis, the shortest and longest physician practices after they completed their residency or professional training were 1 and 40 years, respectively, with an average of 18.5±8.2 years. The shortest and longest periods of their independently engagement in respiratory interventions were 3 months and 35 years, respectively, with an average of 10.9±8.3 years. More than half of the surveyed doctors (55.0%, 191/347) had independently performed respiratory endoscopy for 5-15 years, 79 doctors (22.8%) for less than 5 years, and 77 doctors (22.2%) for more than 15 years. As for training, 48.4% of the responding endoscopists attended the specialized training courses. To note, only 23 surveyed hospitals (6.6%) could perform the training of a bronchoscopy trainee.

#### Nursing staff

**Figure 1:**
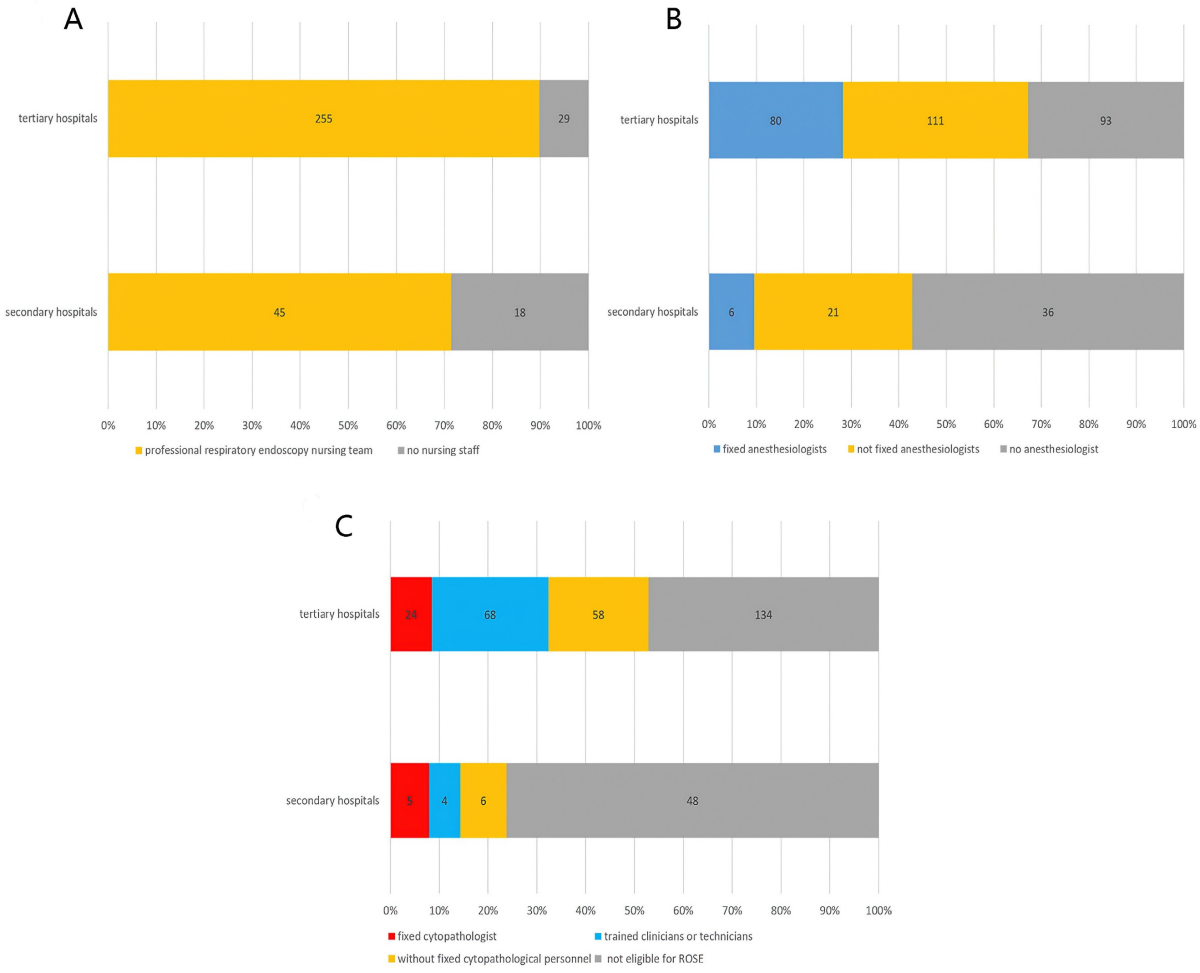
Allocation of respiratory endoscopy staff in different hospitals. **A.** Nursing staff (*χ^2^*=14.843, *P*<0.001); **B.** Anesthesiologist (*χ^2^*=15.936, *P*<0.001); **C.** Cytopathologic staff (*χ^2^*=19.302, *P*<0.001).

Most of the surveyed hospitals (86.5%, 300/347) had a fixed professional respiratory endoscopy nursing team. Of the 47 hospitals that had not, 29 were tertiary general hospitals and 18 were secondary hospitals. The proportion of hospitals without a fixed nursing team was 28.6% (18/63) in secondary hospitals and 10.2% (29/284) in tertiary hospitals (Figure 1A). The difference between tertiary and secondary hospitals is statistically significant (*χ2*=14.843, P<0.001). All of the eight chest or pulmonary specialist hospitals had fixed endoscopic nursing teams as expected.

#### Anesthesiologist

Two hundred and eighteen hospitals (62.8%) staffed with anesthesiologists for respiratory interventions. Among them, the anesthesiologists of 86 hospitals were fixed, accounting for 24.8% of all involved hospitals, and the other 132 hospitals (38.0%) were not fixed. Respiratory endoscopists in 129 hospitals (37.2%) were unable to get the cooperation of an anesthesiologist and they had to anesthetize the patients themselves when needed. As shown in Figure 1B, there is a significant difference in the staffing of anesthesiologists between tertiary hospitals and secondary hospitals (*χ^2^*=15.936, P<0.001).

#### Cytopathologic staff

There were 165 hospitals (47.6%) eligible for rapid on-site evaluation (ROSE) during bronchoscopic biopsy procedures. ROSE was routinely performed by fixed staff in 101 hospitals (29.1%), of which in 29 hospitals (8.4%) was performed by fixed cytopathologists and in the other 72 hospitals (20.7%) by trained clinicians or technicians. ROSE was only performed when needed in 64 hospitals (18.4%) without fixed cytopathological personnel. Figure 1C shows the cytopathologic staff in different levels of hospitals. The difference between tertiary and secondary hospitals is statistically significant (*χ^2^*=19.302, P<0.001).

### Application of diagnostic bronchoscopy procedures in 2021

Percutaneous biopsy for PPLs diagnosis was preferred in 63.4% of the surveyed hospitals, while in the other 127 hospitals (36.6%) bronchoscopy-guided biopsy was believed the first choice for such lesions.

**Figure 2:**
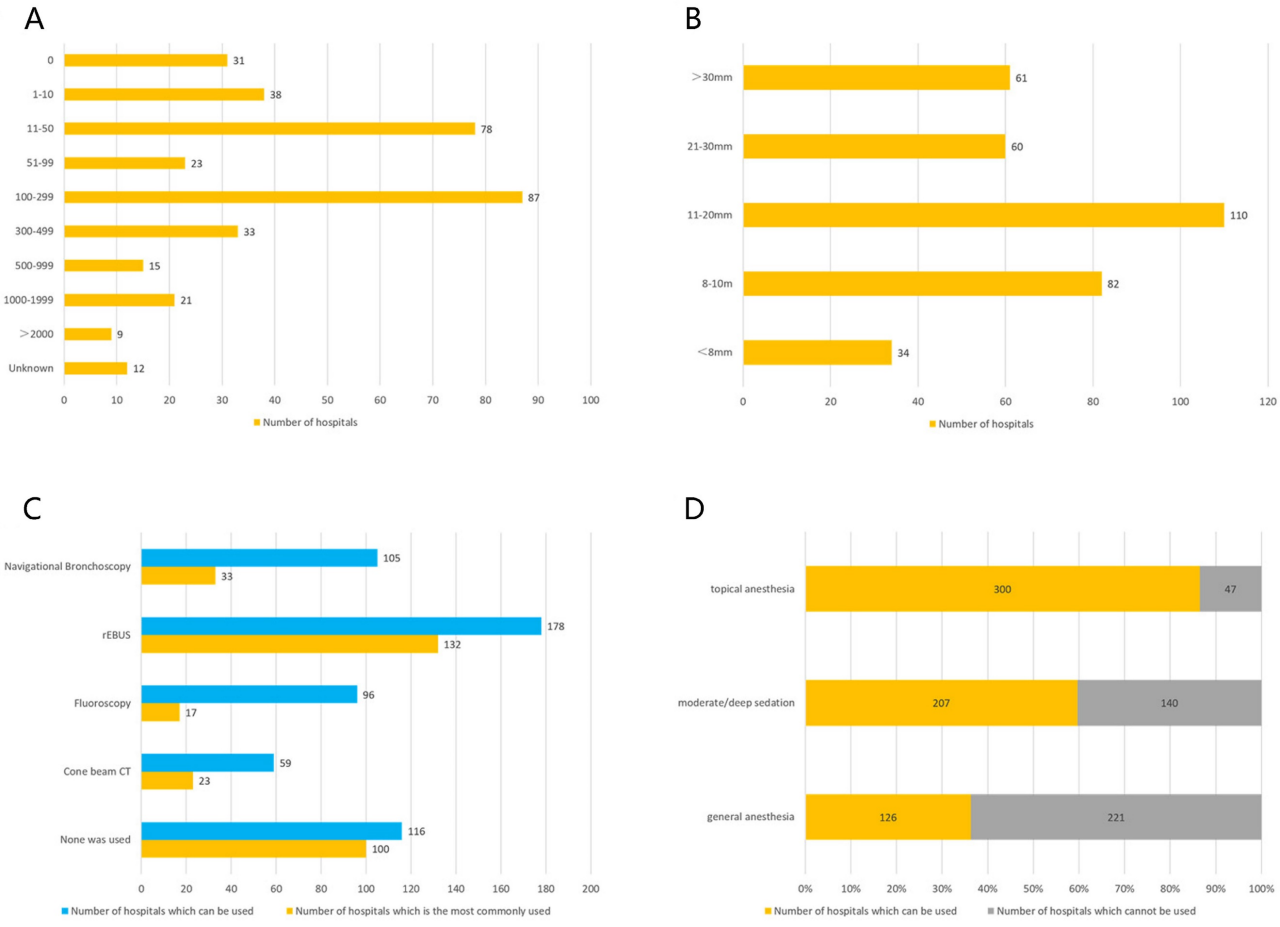
Application of diagnostic bronchoscopy for peripheral pulmonary lesions (PPLs).** A.** Number of PPLs interventional biopsies performed per hospital per year; **B.** Number of hospitals capable of performing transbronchial biopsies of PPLs of different sizes; **C.** Allocated guidance equipments and their applications for diagnostic bronchoscopy; **D.** Anesthesia for diagnostic bronchoscopy.

**Figure 3:**
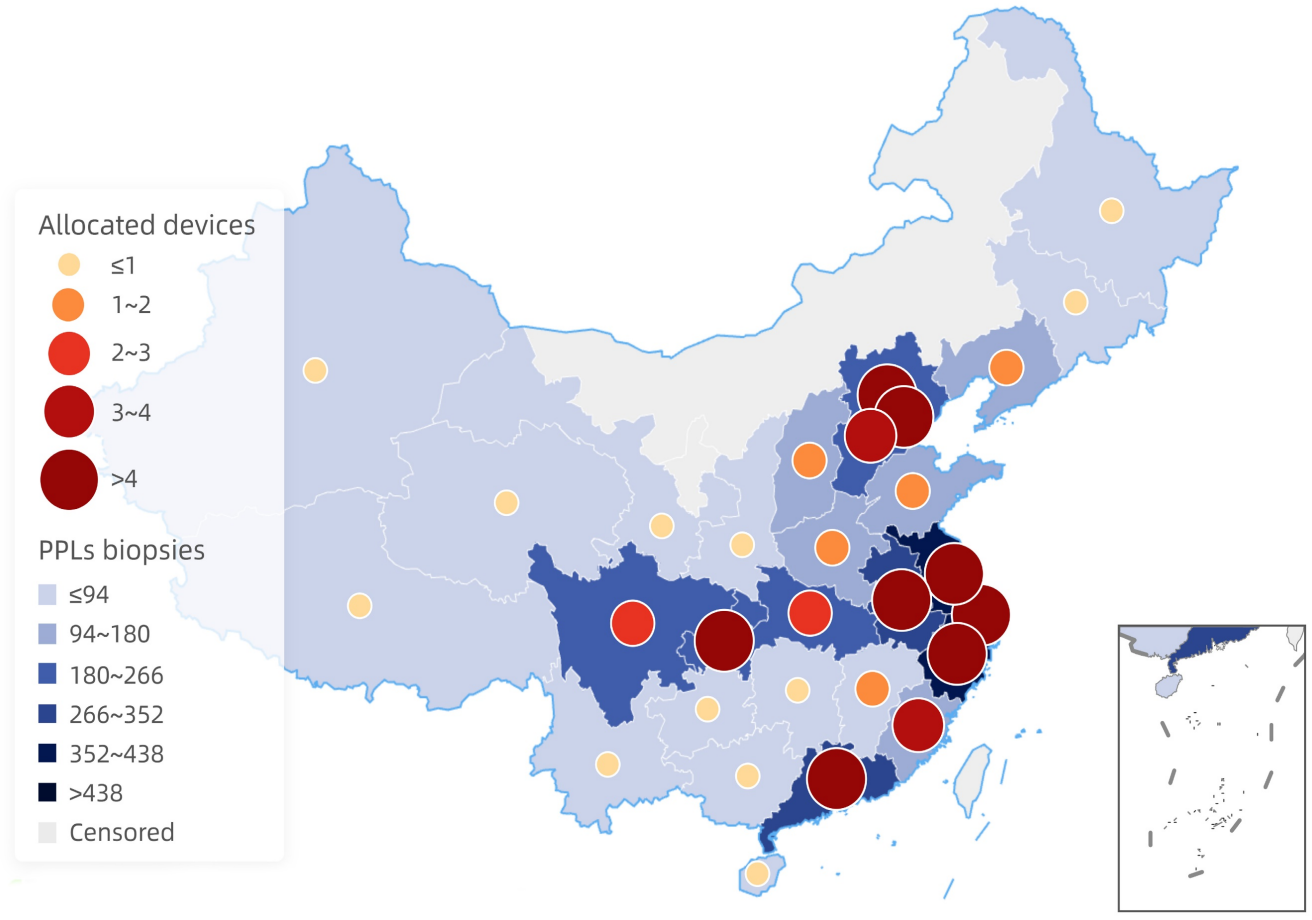
Geographical distributions of PPLs biopsies and allocated devices.

There were 316 hospitals eligible for performing biopsies of PPLs less than 30mm. No more than 10 PPLs were diagnosed by interventional techniques in 38 hospitals per year, 10-50 PPLs biopsies were performed in 78 hospitals per year, 51-99 PPLs biopsies in 23 hospitals, 100-299 PPLs biopsies in 87 hospitals, 300-499 PPLs biopsies in 33 hospitals, 500-1,000 PPLs biopsies in 15 hospitals, 1,000-2,000 PPLs biopsies in 21 hospitals, and more than 2,000 cases in 9 hospitals per year. The remaining 12 hospitals answered "unclear" to this question (Figure 2A). Regarding geographical distributions, performing biopsies of PPLs tended to gain more popularity in the southeast region and coastal cities (Figure 3).

#### Sizes of PPLs diagnosed by bronchoscopy

Thirty-four respondents (9.8%) thought that the minimum diameter of PPLs that could be bronchoscopically biopsied was less than 8 mm, 82 (23.6%) thought it was 8-10 mm, 110 (31.7%) thought it was 11-20 mm, and 60 respondents (17.3%) considered 21-30mm. Sixty-one respondents (17.6%) considered that only PPLs with a diameter of more than 30 mm should be subjected to bronchoscopic biopsy (Figure 2B).

Notably, physicians at all of the involved 347 hospitals responded to this item, although many of them did not actually perform bronchoscopic biopsies of PPLs. We believe that these results should be the choices made by the doctors based on their theoretical viewpoints rather than practical experiences.

#### Guidance techniques for diagnostic bronchoscopy

A variety of techniques were used for the guidance of a bronchoscope to PPLs. According to our survey, r-EBUS was used in 178 hospitals (51.3%) and in 132 hospitals of them it was used as the preferred guidance technique for PPLs biopsies. Other guidance techniques used included navigational bronchoscopy (VBN and ENB) (used in 105 hospitals (30.3%), of which in 33 hospitals (31.4%) was used as the first choice), cone beam CT (CBCT) (used in 59 hospitals (17.0%), of which in 23 hospitals (39.0%) it was used as the first choice), and fluoroscopy (used in 96 hospitals (27.7%), of which in 17 hospitals (17.7%) was used as the first choice). One hundred and sixteen hospitals (33.4%) owned none of the above guidance devices. Bronchial branch tracing that didn't require any extra equipment was the preferred transbronchial guidance technique in 42 hospitals (12.1%), although only 18 hospitals of them did not own any of the above technique and equipment. Additionally, like biopsies of PPLs, the areas with the higher mean number of guidance devices in each hospital were also located in the southeast region and coastal cities (Figure 3), and there was a significant positive correlation between the number of performing PPLs biopsies and the allocated equipment based on Pearson's rank correlation analysis (r = 0.978, P < 0.001). What's more, none of the guidance modalities was used for PPLs biopsies in 100 hospitals (28.8%), even though at least one guidance device was equipped in many of these hospitals (Figure 2C).

#### Navigational bronchoscopy

The equipment and application of six bronchoscopic navigation systems were surveyed. Of the 150 hospitals (43.2%) that owned at least one surveyed navigation system, 114 hospitals owned only one system, 20 hospitals owned 2 systems, 9 hospitals owned 3 systems, 5 owned 4 systems, and one each had 5 and 6 systems. The share ratio of each system was as follow: LungCare ENB® 16.1%, LungPro® 14.4%, SuperDimension® 13.3%, IG4® 6.6%, LungPoint® 5.8%, and LungPoint Plus® 4.9%.

However, navigational bronchoscopy systems were not actually used very often for PPLs biopsies in 30.0% of these hospitals. "High cost" was deemed the main reason for its low utilization by 248 respondents (71.5%). Other reasons attributed to included "low efficiency" (39 responses, 11.2%), "low diagnostic yield" (32 responses, 9.2%), "insufficient professional staffing" (21 responses, 6.1%) and "procedure-related risks and complications” (7 responses, 2.0%).

#### CBCT

CBCT is useful for the application of Navigational bronchoscopy. However, 204 respondents (58.8%) did not have access to CBCT, 125 (36.0%) had occasional access, and only 18 (5.2%) had regular access to the device.

#### Robotic bronchoscopy

Only 3 doctors were "very familiar" with advanced robotic bronchoscopy technique, accounting for 0.86% of all the respondents. The numbers of doctors who chose "know, but not familiar" and "not know at all" about robotic bronchoscopy were 215 (62.0%) and 129 (37.2%).

#### Anesthesia

As presented in Figure 2D, three hundred hospitals (86.5%) applied topical anesthesia by nebulizer with nebulized 4% lidocaine in 10 ml increments before diagnostic bronchoscopy. Moderate/deep sedation and general anesthesia were applied in 207 (59.7%) and 126 hospitals (36.3%), respectively.

Among the 247 hospitals that applied guided-bronchoscopic biopsy for PPLs, 75 hospitals (accounting for 30.4%) "always" or "most of the time" applied general anesthesia when performing such operations. The procedures were performed under sedation or topical anesthesia most of the time in the other hospitals.

### Therapeutic bronchoscopic interventions

Therapeutic bronchoscopic interventions for peripheral lung cancer and/or high-risk PPLs could be performed in 124 of the 347 involved hospitals, accounting for 35.7%. Regarding the question “How to evaluate the application value of bronchoscopy in the treatment of PPLs?”, the vast majority of respondents (210 doctors, 60.5%) chose “very valuable”, 121 (34.9%) chose "limited value", 2 (0.6%) chose "no value", and the remaining 14 (4.0%) thought it "indeterminate".

Compared with percutaneous interventional therapy, 200 doctors (57.6%) believed that bronchoscopic interventions had better application prospects and were more inclined to choose transbronchial interventions for PPLs treatment. There were 133 doctors (38.3%) preferred percutaneous intervention, 6 doctors (1.7%) considered neither method suitable, and 8 (2.3%) were not sure about this issue.

## Discussion

Bronchoscopy was initialized in China from 1970s and the field of interventional pulmonology for PPLs diagnosis and treatment is evolving rapidly 17. The present study revealed that although most of the surveyed doctors could perform interventional biopsies of PPLs, percutaneous biopsy was preferred in two-thirds of the surveyed hospitals instead of less invasive bronchoscopy-guided biopsy. In addition, there was a more than 200-fold difference in the number of PPLs interventional biopsies per year between the most and the least capable hospitals. This unbalanced situation is related to various factors, such as the class and specialty of a hospital, the economic status of the region where a hospital is located, and whether a hospital attaches importance to the development of respiratory endoscopy technology or not, et al. Only when the personnel, equipment, and technical capabilities are all qualified, diagnostic and therapeutic bronchoscopic interventions for PPLs can be performed. We analyzed the situation of the surveyed hospitals in these three aspects.

In addition to endoscopists, a complete high-level respiratory intervention team should consist of fixed professional endoscopic nurses, experienced anesthesiologists and cytopathologists. Most respiratory endoscopists are respiratory physician or thoracic surgeons in China. Professional respiratory endoscopic nurses were available in most hospitals (86.5%). However, there was a shortage of professional anesthesiologists and cytopathologists in many hospitals. The perfect anesthetic staff provides patient comfort and patient safety, while allowing the interventional pulmonologists complete access to the airways and subsequent procedures 21. Some routine diagnostic bronchoscopy procedures (such as forceps biopsy, brushing, bronchoalveolar lavage, et al) can be performed under topical anesthesia or minimal/moderate sedation. For high-risk patients or difficult interventional procedures, deep sedation or general anesthesia is needed. However, three-quarters of the surveyed hospitals lacked fixed experienced anesthesiologists when performing respiratory interventional procedures, and more than one-third of hospitals even failed to obtain the cooperation of a professional anesthesiologist. This makes many advanced respiratory interventional techniques unavailable in these hospitals. The shortage of cytopathologists was even more severe. ROSE allowing rapid stain and real-time assessment for direct slides helps to improve the diagnostic yield of bronchoscopy while reducing the duration of the procedure 22. Less than half of the surveyed hospitals were eligible for ROSE during bronchoscopic procedures and most of the personnel engaged in ROSE in these hospitals were trained clinicians or technicians. Only 29 hospitals (8.1%) had a fixed cytopathologist for diagnostic respiratory interventions. The aforesaid results could be attributed to the presence of heavy daily workload for cytopathologist 23. The lack of professional cytopathologists resulted in a relative low diagnostic yield of transbronchial biopsy of PPLs, which may be one of the main reasons why only 36.6% of respondents preferred bronchoscopic biopsy for PPLs.

Multiple advanced bronchoscopic navigation techniques can be used for guiding the transbronchial approach to PPLs located outside the visible range of the bronchoscope to sample cytohistological materials. r-EBUS, performed by inserting the ultrasound miniature probe through the working channel of a flexible bronchoscope, has worked to improve diagnostic yield by providing the internal structure of the lesion, determining its size, location, and depth of penetration. Since Hürter et al. 24 firstly used r-EBUS for the assessment of normal lungs and bronchial carcinomas in 1992, r-EBUS has gained popularity for the identification of PPLs before biopsy and currently, it was the most widely used bronchoscopic guidance technology according to our survey in China. Similar reports were presented in other countries, such as Czech Republic 18 and Portugal 20. Relatively low technical difficulty, moderate cost, and avoidance of radiation to personnel are thought to be the reasons for the popularity of r-EBUS 25. Furthermore, one-third of the surveyed hospitals did not have any bronchoscopic guidance devices such as fluoroscopy, r-EBUS, etc. This is believed another important reason, probably the main reason, why CT-guided transthoracic biopsy for PPLs was preferred in most hospitals 26. VBN and ENB were also commonly used modalities when evaluating PPLs, with a utilization rate of 30.3%, which was higher than that reported in 2017 (20.4%) 17. The increased trend of utilization rate over time objectively reflected the value of VBN and ENB in the management of PPLs. It was noted that bronchial branch tracing method was the preferred endobronchial guidance technique in 42 hospitals (12.1%), with or without guidance devices. Hand-drawn mapping method for bronchoscopic navigation has been validated to be a feasible, simple and economical guiding modality with time-saving and easy preparation for PPLs biopsies, making it a potential surrogate for techniques that require additional guidance devices in the majority of hospitals with cost-benefit consideration 27. Chinese pulmonologists did not know much about the current state-of-the-art robotic bronchoscopy, with less than 1% of the surveyed doctors being "very familiar" with it, and more than one-third doctors having no knowledge of the technology at all. As far as the authors know, there are currently no more than 3 hospitals that have robotic bronchoscopy in China. The application of robotic bronchoscopy in China is just beginning. It can be seen that where various advanced bronchoscopic navigational techniques are currently available for the diagnosis of PPLs, although prospective, the limitations of each technique should be recognized 4. In order to made up for the deficiency of any single technique, a multimodality approach with combined procedures have been emerged and served as an indispensable component of PPLs diagnosis 28. We attempted to investigate the overall situation concerning combined utilization rate; nevertheless, the absence of available data hindered the investigation.

Two thirds of the surveyed hospitals had at least one bronchoscopic guidance devices, about 44.4% of the hospitals had at least one bronchoscopic navigation system. However, the actual utilization of these devices was not high in these hospitals. This could be explained by the limitation of high cost. Therefore, further policy support and constantly innovated technology are warranted for cost reduction, thereby promoting the widespread adoption of bronchoscopy in the diagnostic and therapeutic work of PPLs. Plus, the surveyed endoscopists independently engaged in respiratory interventions varied considerably in experience. It is well established that there is a learning curve for the diagnosis of PPLs using bronchoscopy that improves with case experience 29-31. A structured education has been recommended for improving the operator's individual experience 32, 33. However, fewer than half of endoscopists attended the specialized training courses. In the Germany survey from 2016, this proportion was 62.5 % 19. Remarkably, only 6.6% of surveyed hospitals were capable of performing the training of a bronchoscopy trainee. In view of this, more attention and investment associated with training are imperative to resolve the existing problems for the furtherance of bronchoscopic practice.

As for geographical distributions, diagnostic bronchoscopy for PPLs was more commonly carried out in the southeast region and coastal cities where the local economy was more developed, revealing the differences in area distributions of bronchoscopy in China. Reducing disparities between different regions was our shared objective; however, there is still a long way to go. Furthermore, we have to emphasize that application of diagnostic bronchoscopy procedures pertains to 2021. Whilst related results, especially the number of PPLs biopsies, could be affected by the COVID-19 pandemic, there has been evaluation regarding area distributions, which minimizes the influence of the COVID-19 pandemic.

Therapeutic bronchoscopy for peripheral lung cancer and/or high-risk PPLs has received extensive attention. About 60% of surveyed pulmonologists believed that transbronchial interventions had better application value and development prospects for the treatment of PPLs than percutaneous methods. However, limited by the above factors such as staffing, technology, and cost, it is still too early to develop therapeutic bronchoscopy for PPLs in most hospitals of China. Currently, it only could be performed in a few well-developed and large-scale respiratory intervention centers.

In summary, the results of this survey showed that bronchoscopy for the diagnosis of PPLs was carried out in most hospitals in China. However, due to factors such as personnel, equipment and technical methods, the diagnostic capabilities and yields in different hospitals and regions varied greatly. As for the application of therapeutic bronchoscopy in PPLs, though most doctors believed in its value and good application prospects, currently only in a few hospitals in China could therapeutic bronchoscopic procedures for PPLs be performed.

## Figures and Tables

**Table 1:** Details of questionnaire

Questions	Options*
A. General information	
1. Which institution do you come from?	Please write down the institution name
2. What class is your institution?	①Secondary general; ②Tertiary general; ③Chest or pulmonary specialist
B. Personnel of respiratory endoscopy team	
1. How long have you been in physician practices after you complete your residency or professional training?	( ) months please specify
2. How long do you independently engage in respiratory interventions?	( ) months please specify
3. Do you attend the specialized training courses associated with bronchoscopy?	①Yes; ②No
4. Is your institution eligible for performing the training of a bronchoscopy trainee?	①Yes; ②No
5. A fixed professional respiratory endoscopy nursing team.	①Yes; ②No
6. Anesthesiologists.	①Fixed; ②Not fixed; ③Neither
7. ROSE.	①Yes; ②No
If yes, who will be responsible for ROSE?	①Fixed cytopathologists; ②Trained clinicians or technicians; ③Non-fixed cytopathologists
C. Application of diagnostic bronchoscopy procedures in 2021	
1. Which is the preferred diagnostic measure you choose for PPLs?	①Percutaneous; ②Bronchoscopic
2. Is your institution eligible for performing biopsies of PPLs less than 30mm?	①Yes; How many cases? ( ) cases; ②No
3. Do you perform diagnostic bronchoscopy for PPLs less than 30mm?	①Yes; How many cases? ( ) cases; ②No
4. Do you think what is the minimum diameter of PPLs that can be bronchoscopically biopsied?	①<8mm; ②8-10mm; ③11-20mm; ④21-30mm; ⑤>30mm
5. Do your institution use the guidance technique for PPLs biopsies?	①Yes; ②No
If yes, choose the guidance technique (s) (multiple choice).	①r-EBUS; ②CBCT; ③Fluoroscopy; ④Navigational bronchoscopy; ⑤ Robotic bronchoscopy; ⑥Bronchial branch tracing
If yes, which is the first transbronchial guidance technique?	①r-EBUS; ②CBCT; ③Fluoroscopy; ④Navigational bronchoscopy; ⑤ Robotic bronchoscopy; ⑥Bronchial branch tracing
6. How many guidance devices does your institution possess?	( ) please write down the number
7. If your institution possesses navigational bronchoscopy, please specify the software.	①LungCare ENB®; ②LungPro®; ③LungPoint®; ④SuperDimension®; ⑤IG4®; ⑥LungPoint Plus®
8. Do you think what is the most important factor affecting the widespread adoption of navigational bronchoscopy in the diagnosis of PPLs?	①High cost; ②Low efficiency; ③Low diagnostic yield; ④Procedure-related risks and complications; ⑤Insufficient professional staffing
9. Do you perform diagnostic bronchoscopy for PPLs using CBCT?	①Often; ②Sometimes; ③Never
10. How much do you know about robotic bronchoscopy?	①Very familiar; ②Know, but not familiar; ③Not know at all
11. Choose the method(s) of anesthesia for PPLs patients who undergo diagnostic bronchoscopy (multiple choice).	①Topical anesthesia; ②Moderate/deep sedation; ③General anesthesia
12. Do you apply general anesthesia for PPLs patients when performing guided-bronchoscopic biopsy?	①Always; ②Most of the time; ③Never
D. Therapeutic bronchoscopic interventions	
1. Do you perform therapeutic bronchoscopy for peripheral lung cancer and/or high-risk PPLs?	①Yes; ②No
2. How to evaluate the application value of bronchoscopy in the treatment of PPLs?	①Very valuable; ②Limited value; ③No value; ④Indeterminate
3. Which method is more promising for PPLs treatment?	①Percutaneous; ②Bronchoscopic; ③Neither; ④Indeterminate

Abbreviations: ROSE, rapid on-site evaluation; PPLs, peripheral pulmonary lesions; r-EBUS, radial probe endobronchial ultrasound; CBCT, cone beam CT. * Only choose one unless otherwise specified.

## References

[1] de Koning HJ, van der Aalst CM, de Jong PA (2020). Reduced Lung-Cancer Mortality with Volume CT Screening in a Randomized Trial. N Engl J Med.

[2] Horeweg N, van der Aalst CM, Thunnissen E (2013). Characteristics of lung cancers detected by computer tomography screening in the randomized NELSON trial. Am J Respir Crit Care Med.

[3] Swensen SJ, Jett JR, Hartman TE (2005). CT screening for lung cancer: five-year prospective experience. Radiology.

[4] Kramer T, Annema JT (2021). Advanced bronchoscopic techniques for the diagnosis and treatment of peripheral lung cancer. Lung Cancer.

[5] Lu CH, Hsiao CH, Chang YC (2012). Percutaneous computed tomography-guided coaxial core biopsy for small pulmonary lesions with ground-glass attenuation. J Thorac Oncol.

[6] Callister ME, Baldwin DR, Akram AR (2015). British Thoracic Society guidelines for the investigation and management of pulmonary nodules. Thorax.

[7] Heerink WJ, de Bock GH, de Jonge GJ (2017). Complication rates of CT-guided transthoracic lung biopsy: meta-analysis. Eur Radiol.

[8] Zarogoulidis P, Huang H, Chen W (2022). Radial Endobronchial Ultrasound for Lung Cancer Diagnosis: Tips and Tricks. J Cancer.

[9] Gasparini S, Mei F, Bonifazi M (2022). Bronchoscopic diagnosis of peripheral lung lesions. Curr Opin Pulm Med.

[10] Tian S, Huang H, Zhang Y (2023). The role of confocal laser endomicroscopy in pulmonary medicine. Eur Respir Rev.

[11] Criner GJ, Eberhardt R, Fernandez-Bussy S (2020). Interventional Bronchoscopy. Am J Respir Crit Care Med.

[12] Tian S, Huang H, Hu Z (2022). A narrative review of progress in airway stents. J Thorac Dis.

[13] Bai C, Choi CM, Chu CM (2016). Evaluation of pulmonary nodules: clinical practice consensus guidelines for Asia. Chest.

[14] Fallon J, Medford A (2017). Endobronchial and transbronchial biopsy experience: A United Kingdom survey. Thoracic Cancer.

[15] Horinouchi H, Asano F, Okubo K (2019). Current status of diagnostic and therapeutic bronchoscopy in Japan: 2016 national survey of bronchoscopy. Respir Investig.

[16] Nie X, Cai G, Li Q (2009). Bronchoscopy in China: the Chinese Society of Respiratory Diseases Survey. Chest.

[17] Shi D, Li F, Wang K (2020). The development of bronchoscopy in China: a national cross-sectional study. J Cancer.

[18] Marel M, Votruba J (2022). Bronchoscopy in the Czech Republic in the past 45 years and the state in 2020. Bratisl Lek Listy.

[19] Hautmann H, Hetzel J, Eberhardt R (2016). Cross-Sectional Survey on Bronchoscopy in Germany-The Current Status of Clinical Practice. Pneumologie.

[20] Guedes F, Ferreira AJ, Dionísio J (2022). Pre- and post-COVID practice of interventional pulmonology in adults in Portugal. Pulmonology.

[21] Semmelmann A, Loop T (2022). Anesthesia for interventional pulmonology. Curr Opin Anaesthesiol.

[22] Fassina A, Corradin M, Zardo D (2011). Role and accuracy of rapid on-site evaluation of CT-guided fine needle aspiration cytology of lung nodules. Cytopathology.

[23] Ito T, Okachi S, Ikenouchi T (2021). The Value of Additional Conventional Transbronchial Biopsy in the Negative Results of Rapid On-site Evaluation During Endobronchial Ultrasound With Guide Sheath to Diagnose Small Peripheral Lung Cancer. Technol Cancer Res Treat.

[24] Hürter T, Hanrath P (1992). Endobronchial sonography: feasibility and preliminary results. Thorax.

[25] Ali MS, Trick W, Mba BI (2017). Radial endobronchial ultrasound for the diagnosis of peripheral pulmonary lesions: A systematic review and meta-analysis. Respirology.

[26] Al-Zubaidi N, Soubani AO (2015). Advances in diagnostic interventional pulmonology. Avicenna J Med.

[27] Zhong CH, Su ZQ, Luo WZ (2022). Hierarchical clock-scale hand-drawn mapping as a simple method for bronchoscopic navigation in peripheral pulmonary nodule. Respir Res.

[28] Zheng X, Cao L, Zhang Y (2022). A Novel Electromagnetic Navigation Bronchoscopy System for the Diagnosis of Peripheral Pulmonary Nodules: A Randomized Clinical Trial. Ann Am Thorac Soc.

[29] Roth K, Eagan TM, Andreassen AH (2011). A randomised trial of endobronchial ultrasound guided sampling in peripheral lung lesions. Lung Cancer.

[30] Huang CT, Ruan SY, Tsai YJ (2017). Experience improves the performance of endobronchial ultrasound-guided transbronchial biopsy for peripheral pulmonary lesions: A learning curve at a medical centre. PLoS One.

[31] Shi J, He J, He J (2021). Electromagnetic navigation-guided preoperative localization: the learning curve analysis. J Thorac Dis.

[32] Du Rand IA, Barber PV, Goldring J (2011). British Thoracic Society Interventional Bronchoscopy Guideline Group. British Thoracic Society guideline for advanced diagnostic and therapeutic flexible bronchoscopy in adults. Thorax.

[33] Lee HJ, Corbetta L (2021). Training in interventional pulmonology: the European and US perspective. Eur Respir Rev.

